# Effectiveness of Custom Foot Insoles to Decrease Plantar Pressure: A Cross-over Randomized Trial Study

**DOI:** 10.3390/healthcare10030575

**Published:** 2022-03-20

**Authors:** Israel Casado-Hernández, Ricardo Becerro-de-Bengoa-Vallejo, Marta Elena Losa-Iglesias, Julia Cosín-Matamoros, César Calvo-Lobo, David Rodríguez-Sanz, Daniel López-López, Eva María Martínez-Jiménez

**Affiliations:** 1 Faculty of Nursing, Physiotherapy and Podiatry, Department of Nursery, Universidad Complutense de Madrid, 28040 Madrid, Spain; isracasa@ucm.es (I.C.-H.); ribebeva@ucm.es (R.B.-d.-B.-V.); cescalvo@ucm.es (C.C.-L.); davidrodriguezsanz@ucm.es (D.R.-S.); evamam03@ucm.es (E.M.M.-J.); 2 Faculty of Health Sciences, Universidad Rey Juan Carlos, 28922 Alcorcón, Spain; marta.losa@urjc.es; 3 Research, Health and Podiatry Group, Department of Health Sciences, Faculty of Nursing and Podiatry, Industrial Campus of Ferrol, Universidade da Coruña, 15403 Ferrol, Spain; daniel.lopez.lopez@udc.es

**Keywords:** plantar pressure, baropodometry, insole, motorcycle injury

## Abstract

Background: Harderness insoles decrease plantar pressure and reduce the foot injury incidence in sport. The purpose of our study was to analyze the plantar pressure variation in moto riders after riding in a real speed circuit with a custom foot 52^0^ Shore EVA insole. Methods: A crossover randomized trial study was performed (consent no. #050520165316). Riders were assessed by an expert motorsport senior podiatry. The participants’ mean age was 35 ± 3.29. Participants completed a 20 min training riding with their own motorcycle in a real speed circuit. Plantar pressures were registered with a baropodometric platform evaluating an Ethyl Vinyl Acetate custom foot insole (CFI) manufactured with 3 mm thickness and 52° Shore A hardness. The Plantar pressures were registered before riding, after riding without EVA insole, and after riding with EVA insole. Results: Total Plantar pressures in right and left foot, and total surface area decrease after riding with EVA insoles. Conclusion: The use of an EVA insole with 52^0^ shore A hardness riding on a motorcycle in speed circuit decreased the total plantar pressures and surface areas values.

## 1. Introduction

Motorsport requires a high physical demand in the development of different body positions. To obtain the maximum efficiency in elite and amateur motorcycling depends on a good biomechanical performance [1,2]. Furthermore, a rider, a motorcycle, and tires integrated with the environment are required to realize the best performance by the rider [3]. To improve moto rider performance on a speed track with multiples turns, riders require accuracy in the dynamics of the motorcycle fitting the Center of Mass (CoM) in load transfer, turning, breaking, speed, and steering changes on the footpeg [4]. There are scant research data about the influence of the foot posture on the footpeg in driving motorcycle development. A moto rider questionnaire was carried out to analyze the discomfort riding on a motorcycle and most of the surveyed riders commented that despite that the foot pain reduced their performance, they could keep riding the motorcycle [5].

Riders have postural and biomechanical variations on the motorcycle when they rest their feet on the footpeg, causing an alteration in the ankle muscles [6], as well as modifications of the sensory information from the hip and knee joint of the leg that supports more force on the footpeg [7].

Scientific literature has shown the influence of different hardness of insoles in the plantar pressure distribution in the motorcycle feet, concluding that the use of hard insoles with aluminum in metatarsal heads decreases forefoot plantar pressure and is more comfortable [8]. On the other hand, recent scientific studies about moto rider musculoskeletal activity in lower limbs and riding on an elite Supersport moto simulator concluded that the use of harder insoles decreased the electromyographic peak activity patterns in all the thigh and hip muscle and all the lower limb muscle except the fibularis longus, due to contact of the knee with the ground in the curve step [9,10].

Recent studies have demonstrated the usefulness of the baropodometric platform to diagnose several diseases on plantar pressure [11,12,13,14]. Feet are essential in the stability and propulsion of the human being [15]. The main foot function in the human body is to transfer body weight, in static and dynamic positions, generating stability and flexibility [16]. Biomechanically, feet are used for posture maintenance and symmetrical distribution of plantar pressure responsible [17,18,19].

The purpose of this research was to analyze the load and distribution variation of the plantar pressures in moto riders using different hardness of insoles after riding on a speed circuit.

## 2. Material and Methods

### 2.1. Design and Sample

The sample size for the study was nine healthy amateur moto riders from an expert moto rider group composed of 35 subjects, recruited while training on a speed circuit in Madrid (Spain) by a foot specialist in September 2020 applying a random, consecutive sampling method. Subjects wore their own riding clothes on the speed circuit, including specific riding boots that fit the different insoles. All the participants had to meet the inclusion criteria: a general check-up was performed and healthy subjects without musculoskeletal injuries, foot and ankle disorders (hallux abductus valgus, hallux limitus, and ankle instability), or neurological alterations affecting the spine or lower limbs were included. With all this, all subjects were eligible to undergo the study. No participants had worn custom orthotic insoles previously. Inclusion in the study was voluntary and each study subject had to read and sign an informed consent form with detailed information in comprehensive language about the investigation to be carried out, the effect of the intervention, and the possibility of renouncing participation at any time during the study.

We performed a cross-over randomized trial study. The University Rey Juan Carlos of Madrid ethics committee authorized this study (consent no. #050520165316). Informed consent was obtained from all subjects prior to their intervention. Our report follows the guidelines and checklist in the Template for Intervention Description and Replication (TIDieR) [20].

Software from The Unidad de Epidemiología Clínica y Bioestadística, Complexo Hospitalario Universitario de A Coruña, Universidade A Coruña calculated the sample size (www.fisterra.com (accessed on 3 September 2020)). Our goal was to test for differences in peak pressure similar to previous research in cyclists’ feet using prefabricated insoles with Ethylene-Vinyl-Acetate (EVA) material. The mean forefoot pressure using flat and outlined insoles in hallux was 5.06 ± 1.19 N/cm^2^, in the first metatarsal head was 3.73 ± 0.84 N/cm^2^, in the second and third metatarsal head 3.82 ± 0.64 N/cm^2^, and the fourth and fifth metatarsal head 3.18 ± 1.10 N/cm^2^, respectively [8]. To achieve this with statistical confidence, an 80% statistical power analysis (*β* = 20%, *α* = 0.05, two-tailed test) with a sample of eight subjects was required.

Nine male subjects took part in the study. A total of 18 feet were analyzed. All subjects were trained to compete in speed circuit with motorcycle. All the participants were trained to an expert level of riding on the speed circuit. Participants performed similar lap times in the training speed circuit to those with a riding experience more than 3 years. The mean age of the participants was 35 ± 3.9 (28–39) years, mean height was 175 ± 0.05 (164–180) cm, mean weight was 73 ± 5.97 (62–82) kg and body mass index (BMI) was 23.99 ± 1.11 (21.97–25.31). All Demographic data are displayed in Table 1.

### 2.2. Procedure

To start, a senior podiatrist, with 15 years specializing in motorsport, assessed each rider with a complete physical clinical evaluation and measured anthropometric variables such as weight, body mass index, and height.

The protocol began with a pre-riding plantar pressure evaluation on the baropodometric platform. For the plantar pressure evaluation, subjects were requested to stay in a standing position on a baropodometric pressure platform (Medicapteurs, Balma, France) [21,22]. The baropodometric size platform was 530 mm length, 600 mm width, and 45 mm height. The thickness was 4 mm, and the platform had an active surface of 400 × 400 mm. The platform sensor specifications were a thickness of 0.15 mm, calibrated resistive, and 8 × 8 mm size. The number of sensors was 2304 and the sensor pressure minimum was 0.4 N/m^2^ (0.0004 kPa) and sensor maximum pressure 100 N/m^2^ (0.1 kPa). Data were recollected by a laptop with a USB interface to the baropodometric platform. Data acquisition frequency was 200 images per second and the vertical force recording was at 60 Hz. Pressure sensor measurements from the baropodometric platform were precise to the nearest 0.001 kg/cm^2^. Before each utilization, auto-calibration was accomplished.

Before testing was conducted, riders took a warm-up lap on their motorcycle. During this time, riders drove on the motorcycle to adjust to the environment and ensure the shoes were comfortable. Each rider wore their own boots with the original pre-made insole.

The test was performed in the Jarama speed circuit in Madrid. The speed circuit of the Jarama is composed of 13 turns, eight right turns and five left turns, with a total circuit length of 3850 m. Riders completed the round at approximately 270 km/h speed on the straightaways and at decreased speeds in the turns over a period of 20 min riding on the speed circuit on the motorcycle for each insole testing. All participants signed an informed consent. The insole selection was random and blind. All riders rode normally on the speed circuit and none showed or made changes in their performance.

We evaluated a non-contoured EVA custom foot insole (CFI) manufactured by an expert orthopedic with 12 year’s experience. From a 3 mm thickness EVA sheet with 52° Shore A hardness, several insoles with different foot measurements (range from 36 to 47 european size foot) with the standard foot contour shape were manufactured. Later, each EVA insole was ensured to fit in the participant boots with no discomfort. Hardness was measured with a durometer (durometer model Vickers PCE-1000, PCE Ibérica S.L. Tobarra, Albacete, Spain). We tested each rider with their own boot insole and with the EVA CFI. The riders’ testing order was randomized and was the same across all riders, and the testing insoles were outlined.

The control plantar pressure was measured before riding with participants standing on the baropodometric platform with bare feet. The static plantar pressure assessment was used and riders were instructed to stand barefoot on the baropodometric [22]. The foot was placed at 15° degrees from midline being the total degree feet separation 30° [23,24]. The upper limbs of the participants were placed in a comfortable position on the body [23]. The participants were requested to stand for 30 s with their eyes open looking on a point at eye level 2 m away [25].

Surface area and foot plantar aera on a baropodometric platform were measured during bipodal standing. The rearfoot and forefoot areas surfaces were analyzed separately. For analysis data, the average of three trials were recorded and used.

All analyses were calculated for normality of the distribution using Shapiro–Wilks, and a distribution was considered normal if the *p* value was major or equal to 0.05.

Furthermore, the demographic quantitative variables (1) age, (2) BMI, (3) height, and (4) weight, were reported with standard deviation (SD), mean, and 95% confidence interval used to explain the findings.

Intra-trial reliability was settled down by ending three records for each rider in each session. The Intra-class correlation coefficients (ICC) were analyzed to evaluate reliability among trials in each rider, and the mean scores were calculated as well as the standard error of measurement (SEM) [26].

The interpretation for ICC values were categorized as poor (ICC lower than 0.40), fair (ICC from 0.40 to 0.59), good (ICC from 0.60 to 0.74), and excellent (ICC from 0.75 to 1.0) [27]. Thus, as proposed by Portney and Watkins [28], clinical values with reliability coefficients more than 0.90 improves the probability of the mensuration available.

The SEM for each variable were reported with percentage of the mean (SEM%) proposed by Bland and Altman as follows: SEM is derived from ICC and SD: SEM = SD * sqrt (1—ICC) and SEM % = SEM/mean * 100 % [29].

Each plantar pressure analysis was determined based on Student’s *t*-test for independent samples and a Chi-Square analysis was used to determine differences of insoles. Additionally, the effect size estimation was calculated using the formula ($d=2t/\sqrt{gdl}$), and evaluated by SD of the participants variables. The Cohen’s d size effect test was based as follows: (1) slight (*d* <= 0.20), (2) fair (*d* = 0.20–0.49), (3) moderate (*d* = 0.50 a 0.79), and (4) large (*d* > 0.80) [30].

In agreement with the determination of the normality of each variable, paired Student *t* tests were used for parametric data and paired samples Wilcoxon test were used for non-parametric data in order to contrast the findings along the completed follow-up.

The student-*t*-tests were used for independent samples in order to define if differences among groups were statistically significant for parametric data, and Mann–Whitney *U* tests were calculated to determine if differences among groups were statistically significant for non-parametric data.

Finally, the *p* value (<0.05) based on an interval of confidence of 95% was defined statistically significant for all tests, using the software called SPSS 19.0 for Windows (IBM, Chicago, IL, USA).

## 3. Results

### 3.1. Sociodemographic Riders Characteristics

Anthropometric and sociodemographic characteristics of the motorcyclists are shown in Table 1.

### 3.2. Baropodometric Normality Values for Plantar pressure and Surface with Different Insoles before and after Riding

All the variables in Table 2 had a normal distribution (*p* > 0.05) except Total Surface Area (cm^2^) Control Right Foot (*p* = 0.012), Total Surface Area (cm^2^) After Riding EVA Left Foot (*p* = 0.007), Total Surface Area (cm^2^) After Riding EVA Right Foot (*p* = 0.048), Total Plantar Pressure (Kpa) After Riding EVA Left Foot (*p* = 0.047), Rearfoot Maximum Peak Pressure (Kpa) After Riding Right Foot (*p* = 0.017), Total Percentages Plantar (%) After Riding EVA Left Foot (*p* = 0.000), Total Percentages Plantar (%) After Riding EVA Right Foot (*p* = 0.001), Forefoot Percentages Plantar (%) After Riding EVA Left Foot (*p* = 0.000), and Rearfoot Percentages Plantar (%) After Riding EVA Left Foot (*p* = 0.010) variables.

### 3.3. Baropodometric Intraclass Correlation Coefficients Values for Different Insoles before and after Riding in Speed Circuit on Motorcycle

The ICC values performed in right and left foot before riding on a Supersport motorcycle, after riding on a Supersport motorcycle, and after riding with an EVA insole on a Supersport motorcycle are shown in Table 3. All ICC values were excellent, only the Rearfoot Percentages Plantar (%) Control Right Foot variable had a fair value.

Table 4 shows the maximum plantar pressure and surface descriptive values measured with baropodometric platform performed on the right and left foot before riding on a Supersport motorcycle, after riding on a Supersport motorcycle, and after riding with an EVA insole on a Supersport motorcycle.

The baropodometric reliability values in Table 3 were statistically significant in all variables after riding with EVA less rearfoot Percentages Plantar (%) Control Right Foot (*p* < 0.05). Means values in Table 4 decreased in all variables after riding with the EVA insole compared with the control and after riding variables. The total plantar pressure in the left and right foot were statistically significant after riding with EVA insole compared with other variables (*p* < 0.05). Forefoot maximum peak pressure (*p* < 0.05) after riding using EVA insoles was statistically significant compared with the control.

## 4. Discussion

The main support points on the motorcycle are the hands on the handlebar and the feet on the footpegs. Riders’ gestures and biomechanical positions of high speed riding when cornering, braking, and changing direction, and the load of the specific sports clothing, such as helmet, leather suit, and boots, may combine to generate a great load on the riders’ feet on the footpegs [31].

Several studies have highlighted the importance of wearing specific sportswear to avoid injuries in riders [32,33] and the relevance that preventive behavior, such as power training, can reduce the musculoskeletal injury riders risk [2]. In addition, previous research about the plantar pressure in moto riders maintains that harder insoles reduce plantar pressure [8]. That research was realized using an elite moto simulator reproducing the same biomechanical movements on the simulator. In our research, the study was realized in a real speed circuit with Supersport motorcycles where riders performed multiple bends and turns, requiring precise control of the dynamics of the motorcycle from the rider. Inertial forces and the environmental variables to which riders were required in the performing biomechanical riding, influenced in our research. The mean plantar pressures values decrease after riding using the EVA insole compared with the control and after riding without EVA insole.

In other hand, the mechanical motorcycle influenced in our research. Gear change performance in motorcycles was carried out with the left foot; to engage the first gear, the lever located at the feet is operated upwards and the following gears are operated downwards, generating a greater plantar support.

The rear brake is located on the right foot, and, to brake, the rider has to perform plantar flexion generating more plantar pressure. There was a statistically significant in the total plantar pressure using EVA insoles after riding in the right and left foot and the mean values comparing left and right foot were higher in the left foot than right foot due to the increased demand in changing gears constantly. The study conducted by Casado et al. concluded that the use of polypropylene insoles with aluminum in metatarsal heads decreased pressure, but the limitation in his research was the influence of the inertial forces, environmental factors, and the influence of changing gears in a real speed circuit [8].

The amount of plantar pressure was statistically significantly reduced after riding with CFI-EVA insoles, showing a decrease of 77.48 and 139.23 Kpa for total plantar pressure of the left and right foot, respectively, a decrease of 41.52 Kpa for Forefoot Maximum Peak Pressure of the right foot, decrease of 78.04 and 131.78 KPa for rearfoot maximum peak pressure of the left and right foot, respectively, and an increase of 24.04 Kpa for the forefoot maximum peak pressure for left foot. The Jarama speed circuit is composed of more right corners than left corners. The increase of the forefoot maximum peak pressure can be caused by the decrease in the contact time of this foot in the course of the circuit producing a shorter foot contact time with the EVA insole. The reported differences support the SEM and MDC, and, thus, were secondary to the use of the proposed insole. These findings were in line with prior studies of our research group, indicating that the use of harder insoles decreased plantar pressures to perform motorcycling [8,9,10].

The mean total surface area values decreased after riding using the EVA insole compared to before riding and after riding without EVA insole. Bousie et al. analyzed the effect of using contoured insoles in cycling and the results indicated an increase in the contact foot area [34] after using contoured insoles. Flat insoles were used in our research, decreasing the plantar surface area.

A limitation in our study is the type of design, where the inclusion of a control group that would improve the findings of this research. Another limitation is associated with influence in plantar pressure and surface plantar area using harder insoles than EVA, such as polypropylene and polypropylene with aluminum in metatarsal heads, employed in previous research [8,9,10]. Additional studies could include this harder insole and would be interesting for future work into the effect of aluminum in metatarsal heads. The present study only measured plantar pressure after riding with insoles. Further studies should analyze plantar pressure during motorcycling in order to describe the standard error of measurement and minimum detectable change of sensor insoles to assess plantar pressure during motorcycling according to a prior study published by our research group.

## 5. Conclusions

The use of an EVA insole with 52^0^ shore A hardness riding on a motorcycle on speed circuits decreases the total plantar pressures and surface areas values. Forefoot maximum peak pressure before riding versus after riding with EVA insole were statistically significant. The findings in this research offer valuable insight for high performance riders to avoid potential and specific musculoskeletal overload injury.

## Figures and Tables

**Table 1:** Anthropometric and sociodemographic characteristics.

Variables	Mean ± SD (*n* = 9)	Range (Min-Max) (*n* = 9)	95% CI (*n* = 9)
Age (years)	35 ± 3.29	28–39	32.57–37.64
Heigh (cm)	175 ± 0.05	164–180	170.82–179.4
Weigh (Kg)	73 ± 5.97	62–82	69.07–78.26
BMI (Kg/m^2^)	23.99 ± 1.11	21.97–25.31	23.14–24.85

Abbreviations: SD: standard deviation; Min: minimum; Max: maximum; 95% CI: 95% confidence interval; cm: centimeters; Kg: kilograms; Kg/m^2^: kilograms/meter2; BMI, Body Mass Index.

**Table 2:** Baropodometric plantar normality values performed in right and left foot before riding on a Supersport motorcycle, after riding on a Supersport motorcycle and after riding with an EVA insole on a Supersport motorcycle.

Variable	Shapiro–Wilk Test	*p*-Value
Total Surface Area (cm^2^) Control Left Foot	0.893	0.216
Total Surface Area (cm^2^) Control Right Foot	0.779	0.012
Total Surface Area (cm^2^) After Riding Left Foot	0.921	0.397
Total Surface Area (cm^2^) After Riding Right Foot	0.968	0.878
Total Surface Area (cm^2^) After Riding EVA Left Foot	0.761	0.007
Total Surface Area (cm^2^) After Riding EVA Right Foot	0.833	0.048
Total Plantar Pressure (Kpa) Control Left Foot	0.940	0.582
Total Plantar Pressure (Kpa) Control Right Foot	0.905	0.281
Total Plantar Pressure (Kpa) After Riding Left Foot	0.942	0.599
Total Plantar Pressure (Kpa) After Riding Right Foot	0.964	0.839
Total Plantar Pressure (Kpa) After Riding EVA Left Foot	0.832	0.047
Total Plantar Pressure (Kpa) After Riding EVA Right Foot	0.952	0.712
Forefoot Maximum Peak Pressure (Kpa) Control Left Foot	0.930	0.482
Forefoot Maximum Peak Pressure (Kpa) Control Right Foot	0.900	0.253
Forefoot Maximum Peak Pressure (Kpa) After Riding Left Foot	0.878	0.149
Forefoot Maximum Peak Pressure (Kpa) After Riding Right Foot	0.865	0.110
Forefoot Maximum Peak Pressure (Kpa) After Riding EVA Left Foot	0.845	0.066
Forefoot Maximum Peak Pressure (Kpa) After Riding EVA Right Foot	0.982	0.974
Rearfoot Maximum Peak Pressure (Kpa) Control Left Foot	0.896	0.227
Rearfoot Maximum Peak Pressure (Kpa) Control Right Foot	0.916	0.359
Rearfoot Maximum Peak Pressure (Kpa) After Riding Left Foot	0.895	0.226
Rearfoot Maximum Peak Pressure (Kpa) After Riding Right Foot	0.794	0.017
Rearfoot Maximum Peak Pressure (Kpa) After Riding EVA Left Foot	0.930	0.481
Rearfoot Maximum Peak Pressure (Kpa) After Riding EVA Right Foot	0.926	0.447
Total Percentages Plantar (%) Control Left Foot	0.840	0.058
Total Percentages Plantar (%) Control Right Foot	0.840	0.058
Total Percentages Plantar (%) After Riding Left Foot	0.847	0.069
Total Percentages Plantar (%) After Riding Right Foot	0.847	0.069
Total Percentages Plantar (%) After Riding EVA Left Foot	0.609	0.000
Total Percentages Plantar (%) After Riding EVA Right Foot	0.691	0.001
Forefoot Percentages Plantar (%) Control Left Foot	0.865	0.110
Forefoot Percentages Plantar (%) Control Right Foot	0.938	0.560
Forefoot Percentages Plantar (%) After Riding Left Foot	0.837	0.053
Forefoot Percentages Plantar (%) After Riding Right Foot	0.841	0.059
Forefoot Percentages Plantar (%) After Riding EVA Left Foot	0.633	0.000
Forefoot Percentages Plantar (%) After Riding EVA Right Foot	0.897	0.236
Rearfoot Percentages Plantar (%) Control Left Foot	0.878	0.151
Rearfoot Percentages Plantar (%) Control Right Foot	0.934	0.520
Rearfoot Percentages Plantar (%) After Riding Left Foot	0.927	0.457
Rearfoot Percentages Plantar (%) After Riding Right Foot	0.878	0.150
Rearfoot Percentages Plantar (%) After Riding EVA Left Foot	0.775	0.010
Rearfoot Percentages Plantar (%) After Riding EVA Right Foot	0.875	0.090

Abbreviations: EVA, ethylene vinyl acetate; cm^2^, square centimeter; Kpa, kilopascal; %, Percentage; *p* values were from Shapiro–Wilk tests.

**Table 3:** Baropodometric platform Intraclass Correlation Coefficients, Standard Error of Measurement and Minimal Detectable Change values performed in right and left foot before riding on a Supersport motorcycle, after riding on a Supersport motorcycle, and after riding with an EVA insole on a Supersport motorcycle.

Total Surface Area (cm^2^) Control Left Foot	0.980 (0.935–0.995)	1.463	4.057	<0.001
Total Surface Area (cm^2^) Control Right Foot	0.979 (0.901–0.995)	1.585	4.394	<0.001
Total Surface Area (cm^2^) After Riding Left Foot	0.966 (0.869–0.992)	1.908	5.289	<0.001
Total Surface Area (cm^2^) After Riding Right Foot	0.864 (0.567–0.967)	4.034	11.182	<0.001
Total Surface Area (cm^2^) After Riding EVA Left Foot	0.986 (0.952–0.997)	1.224	3.394	<0.001
Total Surface Area (cm^2^) After Riding EVA Right Foot	0.992 (0.942–0.998)	0.978	2.712	<0.001
Total Plantar Pressure (Kpa) Control Left Foot	0.937 (0.765–0.985)	4.515	12.516	<0.001
Total Plantar Pressure (Kpa) Control Right Foot	0.897 (0.690–0.975)	4.297	11.911	<0.001
Total Plantar Pressure (Kpa) After Riding Left Foot	0.995 (0.983–0.999)	1.272	3.526	<0.001
Total Plantar Pressure (Kpa) After Riding Right Foot	0.980 (0.926–0.995)	1.893	5.248	<0.001
Total Plantar Pressure (Kpa) After Riding EVA Left Foot	0.991 (0.974–0.998)	1.706	4.73	<0.001
Total Plantar Pressure (Kpa) After Riding EVA Right Foot	0.990 (0.971–0.998)	1.339	3.711	<0.001
Forefoot Maximum Peak Pressure (Kpa) Control Left Foot	0.841 (0.494–0.961)	9.518	26.382	<0.001
Forefoot Maximum Peak Pressure (Kpa) Control Right Foot	0.985 (0.956–0.996)	2.966	8.222	<0.001
Forefoot Maximum Peak Pressure (Kpa) After Riding Left Foot	0.993 (0.979–0.998)	1.997	5.535	<0.001
Forefoot Maximum Peak Pressure (Kpa) After Riding Right Foot	0.974 (0.919–0.994)	3.905	10.825	<0.001
Forefoot Maximum Peak Pressure (Kpa) After Riding EVA Left Foot	0.946 (0.830–0.987)	5.546	15.375	<0.001
Forefoot Maximum Peak Pressure (Kpa) After Riding EVA Right Foot	0.994 (0.980–0.998)	1.876	5.200	<0.001
Rearfoot Maximum Peak Pressure (Kpa) Control Left Foot	0.922 (0.699–0.982)	2.424	6.719	<0.001
Rearfoot Maximum Peak Pressure (Kpa) Control Right Foot	0.969 (0.905–0.992)	2.121	5.880	<0.001
Rearfoot Maximum Peak Pressure (Kpa) After Riding Left Foot	0.842 (0.486–0.961)	3.450	9.563	<0.001
Rearfoot Maximum Peak Pressure (Kpa) After Riding Right Foot	0.953 (0.858–0.988)	2.612	7.241	<0.001
Rearfoot Maximum Peak Pressure (Kpa) After Riding EVA Left Foot	0.968 (0.901–0.992)	1.552	4.303	<0.001
Rearfoot Maximum Peak Pressure (Kpa) After Riding EVA Right Foot	0.967 (0.897–0.992)	2.188	6.067	<0.001
Total Percentages Plantar (%) Control Left Foot	0.968 (0.904–0.992)	0.273	0.758	<0.001
Total Percentages Plantar (%) Control Right Foot	0.968 (0.904–0.992)	0.432	1.199	<0.001
Total Percentages Plantar (%) After Riding Left Foot	0.978 (0.913–0.995)	0.226	0.629	<0.001
Total Percentages Plantar (%) After Riding Right Foot	0.978 (0.913–0.995)	0.358	0.994	<0.001
Total Percentages Plantar (%) After Riding EVA Left Foot	0.953 (0.856–0.988)	0.331	0.919	<0.001
Total Percentages Plantar (%) After Riding EVA Right Foot	0.953 (0.856–0.988)	0.524	1.454	<0.001
Forefoot Percentages Plantar (%) Control Left Foot	0.805 (0.430–0.951)	0.984	2.729	0.002
Forefoot Percentages Plantar (%) Control Right Foot	0.936 (0.806–0.984)	0.584	1.619	<0.001
Forefoot Percentages Plantar (%) After Riding Left Foot	0.863 (0.580–0.966)	0.825	2.287	<0.001
Forefoot Percentages Plantar (%) After Riding Right Foot	0.886 (0.648–0.972)	0.779	2.161	<0.001
Forefoot Percentages Plantar (%) After Riding EVA Left Foot	0.933 (0.798–0.983)	0.577	1.599	<0.001
Forefoot Percentages Plantar (%) After Riding EVA Right Foot	0.768 (0.315–0.942)	1.112	3.084	0.005
Rearfoot Percentages Plantar (%) Control Left Foot	0.960 (0.877–0.990)	0.474	1.313	<0.001
Rearfoot Percentages Plantar (%) Control Right Foot	0.435 (−0.598–0.856)	2.315	6.417	0.147
Rearfoot Percentages Plantar (%) After Riding Left Foot	0.939 (0.818–0.985)	0.585	1.622	<0.001
Rearfoot Percentages Plantar (%) After Riding Right Foot	0.965 (0.895–0.991)	0.576	1.590	<0.001
Rearfoot Percentages Plantar (%) After Riding EVA Left Foot	0.906 (0.713–0.977)	0.726	2.014	<0.001
Rearfoot Percentages Plantar (%) After Riding EVA Right Foot	0.987 (0.960–0.997)	0.351	0.973	<0.001

Abbreviations: ICC; Intraclass Correlation Coefficient; 95% CI; Confidence Interval; SEM, Standard Error of the Mean; MDC, Minimal Detectable Change; EVA, Ethil Vinyl Acetate; cm^2^, square centimeter; Kpa, kilopascal; %, Percentage; a *p* value < 0.05 with a confidence interval of 95% was considered statistically significant.

**Table 4:** Maximum plantar pressure and surface measured with podobarometric platform performed on right and left foot before riding on a Supersport motorcycle, after riding on a Supersport motorcycle with conventional insoles, and after riding with an EVA insole on a Supersport motorcycle, and comparisons values before and after riding on motorcycle in speed circuit with EVA insoles.

Foot Measurement Variable	Before Riding (Control)		After Riding		After Riding EVA		Before Riding (Control) vs. after Riding	Before Riding (Control) vs. after Riding EVA
	MEAN ± SD (95% CI)	Median IQR	MEAN ± SD (95% CI)	Median IQR	MEAN ± SD (95% CI)	Median IQR		
Total Surface Area (cm^2^) Left Foot	143.70 ± 7.53 (137.91–149.49)	143.00 16	145.55 ± 5.95 (140.97–150.13)	144.66 9.50	134.74 ± 17.57 (121.22–148.25)	123.33 35.16	0.286	0.066
Total Surface Area (cm^2^) Right Foot	146.29 ± 9.58 (138.93–153.66)	153.00 19	154.62 ± 2.83 (152.45 −156.80)	154.00 4.49	137.74 ± 20.42 (122.04–153.44)	145 42.16	0.069	0.138
Total Plantar Pressure (Kpa) Left Foot	825.48 ± 9.68 (818.03–832.92)	825.66 16.66	789.51 ± 24.57 (770.62–808.40)	792.00 37.33	748 ± 19.72 (733.39–762.60)	741.00 3.50	0.008	0.008
Total Plantar Pressure (Kpa) Right Foot	779.74 ± 4.76 (776.74–783.40)	781.00 15.33	692.74 ± 19.04 (678.10–707.38)	690.66 31.50	640.51 ± 16.36 (627.94–653.09)	642 25.00	0.008	0.008
Forefoot Maximum Peak Pressure (Kpa) Left Foot	495.88 ± 6.24 (491.09–500.68)	496.33 17.66	534.14 ± 58.64 (489.07–579.22)	556.00 50.66	519.92 ± 6.73 (514.74–525.10)	517.66 12.16	0.086	0.008
Forefoot Maximum Peak Pressure (Kpa) Right Foot	580 ± 11.95 (570.80–589.19)	582.00 22.66	567.40 ± 46.05 (532–602.80)	557.66 64.66	538.48 ± 14.66 (527.20–549.75)	538.33 22.83	0.553	0.008
Rearfoot Maximum Peak Pressure (Kpa) Left Foot	823.07 ± 9.52 (815.75–830.39)	818.66 16.83	752.14 ± 4.82 (748.42–755.86)	753.00 4.50	745.03 ± 11.71 (736.02–754.04)	741.66 14.16	0.008	0.008
Rearfoot Maximum Peak Pressure (Kpa) Right Foot	774.59 ± 11.42 (765.81–783.37)	773.33 21.83	679.77 ± 13.56 (669.34–690.20)	673.00 27.16	642.81 ± 11.18 (634.22–651.40)	642.33 11.83	0.008	0.008
Total Percentajes Plantar (%) Left Foot	49.92 ± 2.41 (48.07–51.78)	49.00 3.33	50.48 ± 2.49 (48.56–52.39)	51.00 4.00	50.81 ± 2.18 (49.13–52.49)	51.66 1.50	0.765	0.191
Total Percentajes Plantar (%) Right Foot	50.07 ± 2.41 (48.21–51.92)	51.00 3.33	49.51 ± 2.49 (47.60–51.43)	49.00 4.00	49.55 ± 2.37 (47.72–51.38)	48.50 2.75	0.765	0.406
Forefoot Percentajes Plantar (%) Left Foot	23.33 ± 1.22 (22.39–24.27)	23.66 2.66	23.11 ± 1.10 (22.26–23.96)	23.11 3.00	22.81 ± 4.36 (19.46–26.16)	25.00 5.66	0.726	0.635
Forefoot Percentajes Plantar (%) Right Foot	25.37 ± 0.63 (24.88–25.85)	25.33 2.00	25.92 ± 5.13 (21.97–29.87)	27.66 9.33	24.00 ± 3.48 (21.32–26.67)	24.33 4.83	0.058	0.523
Rearfoot Percentajes Plantar (%) Left Foot	29.66 ± 2.06 (28.07–31.25)	29.66 4.00	29.77 ± 2.41 (27.91–31.64)	29.33 4.16	28.44 ± 2.63 (26.41–30.47)	27.33 2.33	0.859	0.057
Rearfoot Percentajes Plantar (%) Right Foot	25.37 ± 0.63 (24.88–25.85)	25.33 2.00	25.92 ± 5.13 (21.97–29.87)	27.66 9.33	24.00 ± 3.48 (21.32–26.67)	24.33 4.83	0.859	0.327

Abbreviations: SD, Standard Deviation; 95% CI, 95% Confidence Interval; EVA, Ethil Vinyl Acetate; IQR, Interquartil Range; cm^2^, square centimeter; Kpa, kilopascal; %, Percentage; a *p* value < 0.05 with a confidence interval of 95% was considered statistically significant.

## Data Availability

Not applicable.
